# Oxidative Stress and Complement Activation in Aqueous Cells and Vitreous from Patient with Vitreoretinal Diseases: Comparison Between Diabetic ERM and PDR

**DOI:** 10.3390/antiox14070841

**Published:** 2025-07-08

**Authors:** Lucia Dinice, Pamela Cosimi, Graziana Esposito, Fabio Scarinci, Andrea Cacciamani, Concetta Cafiero, Luca Placentino, Guido Ripandelli, Alessandra Micera

**Affiliations:** 1 Research and Development Laboratory for Biochemical, Molecular and Cellular Applications in Ophthalmological Science, IRCCS—Fondazione Bietti, 00184 Rome, Italy; lucia.dinice@fondazionebietti.it (L.D.); graziana.esposito@fondazionebietti.it (G.E.); 2 Surgical Retina Research Unit, IRCCS—Fondazione Bietti, 00184 Rome, Italy; pcosimi@gmail.com (P.C.); fabioscarinci@gmail.com (F.S.); andrea_cacciamani@hotmail.com (A.C.); luca.placentino@fondazionebietti.it (L.P.); guido.ripandelli@fondazionebietti.it (G.R.); 3 Anatomic Pathology Unit, Fabrizio Spaziani Hospital, 03100 Frosinone, Italy; concettacafiero@gmail.com

**Keywords:** vitreoretinal diseases, epiretinal membranes, diabetes, proliferative diabetic retinopathy, complement system, reactive oxygen species, lipid peroxides, aqueous, vitreous

## Abstract

Background: Epiretinal membrane (ERM) and proliferative diabetic retinopathy (PDR) belong to the group of vitreoretinal diseases, characterized by impairments at both the retina and the vitreous. The non-diabetic and diabetic forms of ERM (no-dERM and dERM) as well as the PDR are caused by microvascular disorder, which frequently occurs in association with inflammation and oxidative stress. To better characterize no-dERM, dERM, and PDR at the biomolecular level, we compared the expression of inflammatory, oxidative, lipidic peroxidation products, and complement receptors. Methods: Twenty-seven ocular fluids from patients who underwent phaco-vitrectomy were categorized as no-dERM (9, 4M/5F; 70.4 ± 6.4), dERM (6, 3M/3F; 73.2 ± 4.9), and PDR (6, 5M/1F; 63.7 ± 7.4). Six cataracts (CTR; 3M/3F; 77.7 ± 9.0) were collected for internal control of aqueous cells. Results: In aqueous cells, *p65NFkB*, *iNOS*, *Nox1/Nox4*, and *Nrf2* were significantly upregulated, and *Keap1* was downregulated in dERM compared with PDR and no-dERM. In aqueous cells, a significant upregulation for *C3aR1mRNA*, *C5aR1mRNA*, and *CFHmRNA* were observed in dERM. In vitreous, C3a, C5b9, and MDA levels were significantly increased in dERM compared with PDR and no-dERM. Conclusions: Inflammatory and ROS products, as well as *C3aR1/C5aR1* and soluble MDA, appear of great interest, as their expression in aqueous and vitreous might have potential prognostic and therapeutic values.

## 1. Introduction

Epiretinal membrane (ERM) and proliferative diabetic retinopathy (PDR) belong to the vitreoretinal diseases (VRD), a complex group of conditions characterized by impairments at both the retina and vitreous, leading to blindness, and vision loss [1,2,3,4,5]. At the cellular level, ERM is a fibrovascular membrane layered at the interface between the vitreous and the retina, characterized by a long-lasting contraction, frequently observed in ocular inflammatory conditions [3]. PDR, the most advanced stage of diabetic retinopathy, is characterized by extensive retinal ischemia, intense neovascularization, abnormal ERM proliferation with tractional retinal detachment, and subsequent bleeding [4]. Risk factors comprise aging, obesity, cardiovascular diseases, and diabetes [1]. Diabetes represents the main risk factor for both dERM and PDR [5]. Diabetic ERMs (dERM) are often linked to diabetic retinopathy, while the idiopathic form is typically associated with aging (no-dERM) [5].

Hyperglycemia is thought to be the primary cause of oxidative stress in different tissues, although metabolic changes as well as genetic and epigenetic factors can contribute massively [6]. Hyperglycemia-driven oxidative stress can trigger local inflammation, lipid peroxidation, microcirculatory abnormalities, mitochondrial defects, neurogenic inflammation, and cell apoptosis, implicating severe structural and functional neurodegenerative changes [7]. The excessive accumulation of reactive oxygen species and nitrogen species (ROS and RNS) can trigger angiogenesis and impairments in the retinal cell network [8,9,10,11,12,13,14]. Besides the metabolic triggers [15], the abnormal activity of nuclear factors has been demonstrated to correlate with the overproduction of ROS, including the activated activity of nuclear factor kappa-light-chain-enhancer of the activated B cells factor (NFκB) and attenuated activity of nuclear erythroid related factor 2 (*Nrf2*; NFE2L2) in some diabetic diseases [16,17,18,19]. Hyperglycemia-mediated mitochondrial dysfunction has been correlated with the overproduction of ROS and impairments in Kelch-like ECH-associated protein 1 (*Keap1*) and Nuclear Factor (erythroid-derived 2-)-like 2 (*Nrf2*) mediators in diabetic retinopathies [20]. The exacerbation of the entire inflammatory process can also lead to lipid peroxidation, even in case of balanced glycemia due to metabolic memory [21,22,23,24]. ROS can directly mainly damage the polyunsaturated lipids, resulting in the formation of peroxyl free radicals, lipid hydroperoxide, and some secondary products like malondialdehyde (MDA)—the most widely used biomarkers for evaluating oxidative stress [25,26,27]. Increased circulating MDA levels might be a risk factor in people with VRD, as observed in the case of increased circulating and vitreous MDA level in association with oxidative stressors, along with poor antioxidant effects [28,29].

Oxidative stress plays a crucial role in alternative complement system activation and particularly in the pathogenesis of some neurodegenerative ocular diseases such as AMD, prospecting the hypothesis of complement dysregulation throughout alternative complement pathway and VEGF interplays [30,31]. The alternative pathway represents a long-lasting, low-level activation executor (parainflammation), particularly relevant for retinal tissue if triggered by metabolic and epigenetic alterations as well as diabetes-induced oxidative stress [32]. Mainly C3a and C5a participate in the pathogenesis of retinal diseases, either through direct damaging effects or by activating the glial subtypes residing inside the retinal microenvironment [33]. Increased C3, C5a, C9, CFB, and CFI have been detected in PDR vitreous as well as specific polymorphisms of the C5 gene [33,34].

Since ocular fluids might have potential pathogenetic value as a reservoir of mediators released upon eye inflammation [35,36], the aim of the present study is to evaluate the expression of inflammatory, oxidative, and complement (alternative path) actors in aqueous cells and vitreous from patients suffering from no-dERM, dERM, and PDR. All biomolecular analyses were carried out in aqueous cells, by investigating the transcript expression of oxidative stress (*p65NFkB*, *iNOS*, *Nox1*, and *Nox4*, as well as *Keap1* and *Nrf2*; [35]) and complement (*C3aR1* and *C5aR1* receptors and the regulatory factor *CFH* [36]) factors, and in clear vitreous, by assessing the protein expression of C3a and C5b9 fragments and the major lipidomic activator/effector MDA.

## 2. Materials and Methods

The study protocol was approved by the intramural ethical committee (IFO-Bietti, Rome, Italy) (MIRRA-V No 94/20/FB) and performed in accordance with the ethical standards stated in the Declaration of Helsinki. Patients approved the experimental approach and signed the consensus for specimen collection, handling and analysis.

### 2.1. Study Population and SD-OCT Classification

A total of twenty-seven patients (27; 15M/12F; 71.1 ± 8.2 years old) were recruited before therapeutic phaco-vitrectomy and grouped as follows: no-dERM (9 ERM from non-diabetic patients); dERM (6 ERM from diabetic patients); and PDR (6 ERM in patients with proliferative diabetic retinopathy). Finally, an additional group of 6 patients with cataracts (CTR) were used as control for the molecular analysis of aqueous cells. The characteristics of the study population are summarized in Table 1. Before surgery, a full ophthalmic examination was carried out, including anamnesis, funduscopic evaluation, and imaging acquisitions (spectral domain optical coherence tomography; Spectralis SD-OCT ver.1.5.12.0; Heidelberg Engineering, Heidelberg, Germany). Inclusion criteria comprised the iatrogenic (no-dERM) and diabetic form (dERM) of ERM and the PDR, while exclusion criteria were macular holes, macular degeneration, retinal detachment, and choroidal neovascularization. At the time of complete ophthalmological visit for enrollment for the study, dERM and PDR subjects reported that they were affected by type-2 diabetes.

Demographic, clinical, and sample information were collected in patients providing written informed consent, as requested according to the Ethical Committee rules.

### 2.2. Sampling Mode and Preanalytics

Both aqueous and vitreous samples were collected from each patient after signing the written informed consent. The procedure of sampling occurred at the beginning of the standard 25 G pars plana vitrectomy. Untouched fluids were quickly stabilized and delivered to the laboratory for appropriate preanalytical steps. Aqueous (100–200 μL) and vitreous (200–500 μL) samples were quickly centrifuged (2000 rpm for 7 min; Sigma 1–14 microfuge) to separate floating cells from clarified supernatant that was quickly stabilized with protease inhibitors (1 µL/sample; Pierce, Thermofisher Scientific, Milan, Italy), sonicated (VibraCell; Sonics, Newtown, CT, USA) to sprinkle residual cells or free nucleic acids (RNA/DNA), and further centrifuged to remove debris (13,000 rpm/7 min). A spectrophotometer analysis (3 μL) was carried out (N1000, Nanodrop; Celbio, Euroclone S.p.A, Milano, Italy) before producing aliquots devoted to molecular and biochemical analysis. Molecular analyses were performed in aqueous cell pellets, while clear vitreous was assayed for biochemical parameters.

### 2.3. Total RNA Isolation, cDNA Synthesis, and Relative Real-Time PCR Analysis

Aqueous cells were subjected to total RNA extraction (MirVana Paris; Thermo Fisher Scientific Inc., Waltham, MA, USA) kit implementations. Total RNA was rehydrated (11 μL RNase-free sterile MilliQ water; Fisher Molecular Biology, FMB Rome, Italy) and checked for quantity/purity using the Nanodrop platform (>1.8; NaNo program). cDNA was synthesized from pure RNA extracts (50–100 ng) in a LifePro Thermal Cycler (Euroclone, Rome, Italy) by using the GoScript RT mix supplemented with random hexamers (Promega, Madison, WI, USA). Thereafter, cDNA (3 μL for the target gene and 1 μL for the referring gene) was amplified with SYBR-green hot-start PCR master mix (Hydra Taq; Biolab; Biocell, Rome, Italy) in both the 48 Eco™ Real-Time PCR platform (Illumina, San Diego, CA, USA) and the Biorad CFX96™ platform equipped with the CFX Maestro 2.3 DXSE software (Bio-Rad Laboratories, Inc., Hercules, CA, USA). Cycle threshold values (Cq) from normalized samples were used for REST analysis (the REST 384–2006 software [37]). Changes in target gene expression observed in cases vs. controls were provided as log2 expression ratios, considering the H3 referring gene. For validation, amplicons (100–200 bps) were separated in 2.5% agarose gel (mini horizontal apparatus; Bio-Rad), and bands were observed/acquired inside the UVP station (tiff-format images). Primer pairs were synthesized by Eurofin MWG Genomics (www.eurofinsgenomics.eu; last access on 17 April 2025), at least one intron spanning (Primer3 Input ver. 0.4.0), and sequences are reported in Table 2.

### 2.4. Quantitative Analysis of C3a, C5b9, and Malondialdehyde (MDA)

Vitreal aliquots were pre-extracted and used for the assays. All incubation and washing procedures (ready-to-use solutions) were performed according to the manufacturers’ suggestions, with minor modifications. Vitreal C3a and C5b9 (50 μL/sample) were prediluted 1:2 and quantified by using the commercially available C3a and C5b-9 ELISA kits (Cloud-Clone Corp., Houston, TX, USA), including both precoated 96-well plates, standards, and reagents. Absorbance (optical density—OD) values were recorded after plate reading (λ450–λ570 nm), and concentrations were produced from standard curves (assay range: 5000 pg/mL–78 pg/mL for C3a and 80 ng/mL–1.25 ng/mL for C5b-9).

MDA levels were measured in 200 µL vitreous by using the Lipid Peroxidation (MDA) assay kit (ab118970; Abcam, Cambridge, UK). Briefly, vitreal lipoproteins were precipitated by adding phosphotungstic acid solution, mixed by vortexing, and centrifuged to remove protein contamination (13,000× *g*/3 min; Sigma 1–14 microfuge; Sigma-Aldrich, Milan, Italy). Thereafter, the pellets were resuspended on ice with ddH_2_O containing BHT and sonicated before analysis. The developer VII/TBA was added to samples and standards, and finally the reaction mix (containing MDA-TBA adduct) was heated at 95 °C for 1 h. Absorbance (optical density; OD) values were recorded after plate reading (λ532 nm), and concentrations were produced according to standard curves (assay range: 20 μM–100 μM). All OD acquisitions were carried out on a Sunrise platform (Tecan, Männedorf, Switzerland).

### 2.5. Statistics

Continuous variables were reported as mean ± SD in the graphics. Data were first analyzed by the Kolmogorov–Smirnov and the Shapiro–Wilk tests to satisfy the assumption of values coming from a normally distributed population (Prism4.0; GraphPad Software Inc., San Diego, CA, USA). One-way ANOVA analysis was used to compare the biomolecular expression between subgroups. Rest-ANOVA Dunnett’s coupled analysis was carried out for identifying significant changes in data coming from real-time PCR experiments. Relations between subgroups were assessed by using the Pearson rho test. Some statistical analysis were confirmed with the RStudio (RStudio/2023.12.0+369). One-way ANOVA followed by a Tukey–Kramer post hoc was used for comparisons of MDA levels between groups. For significance, * *p* < 0.05, ** *p* < 0.01, *** *p* < 0.001, and **** *p* < 0.0001 and rho > 0.700 with *p* < 0.05, or rho > −0.700 with *p* < 0.05 were considered.

## 3. Results

**Figure 1:**
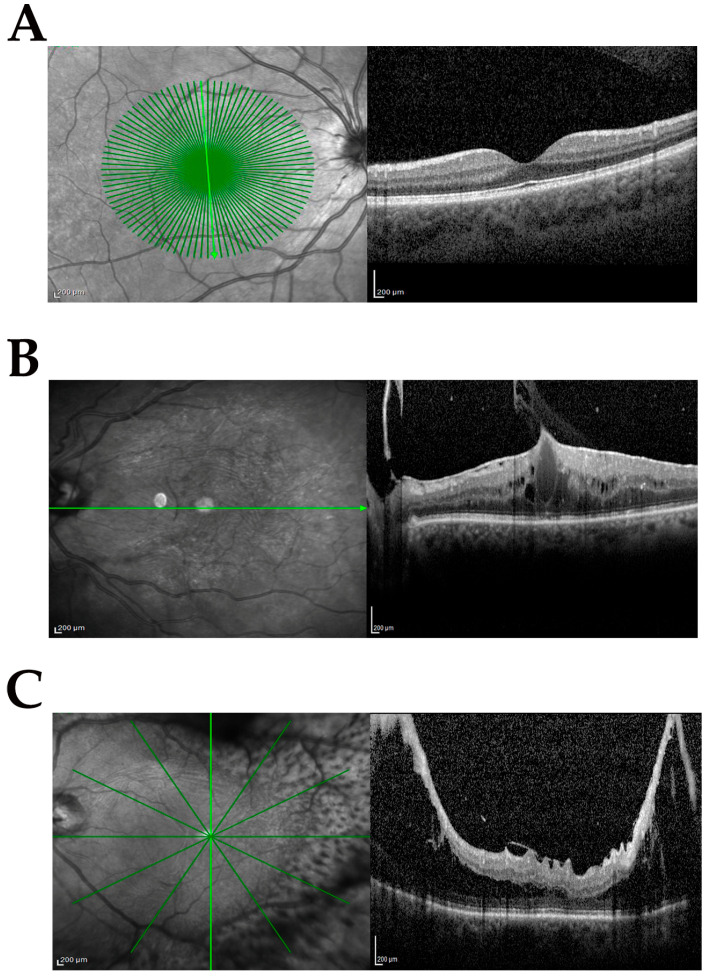
Clinical features at the vitreoretinal interface. Representative infrared (**left**) and related spectral domain optical coherence tomography (OCT, **right**) retinal images from groups investigated: cataract (CTR; (**A**)), diabetic epiretinal membrane (dERM; (**B**)), and proliferative diabetic retinopathy (PDR; (**C**)). Note the cystoid edema and vitreomacular traction in (**B**) and proliferation along the arches causing intraretinal schisis and tractional detachment in (**C**). Green line indicates the region of acquisition; scale bar = 200 µm.

The study population included samples from phaco-vitrectomy comprising control (cataract) and case samples. Three subgroups were produced from the case population according to the following inclusion and exclusion criteria: no-dERM, dERM, and PDR. All subgroups used for this comparative study are shown in Table 1. The complete ophthalmic examination was performed before surgery and disease severity was defined according to standard protocols and used for categorizing biosamples. Figure 1A–C shows the representative SD-OCT images from cataract (Figure 1A), dERM (Figure 1B), and PDR (Figure 1C).

### 3.1. Oxidative Stress Regulators Are Overexpressed in Aqueous-Derived dERM Cells

**Figure 2:**
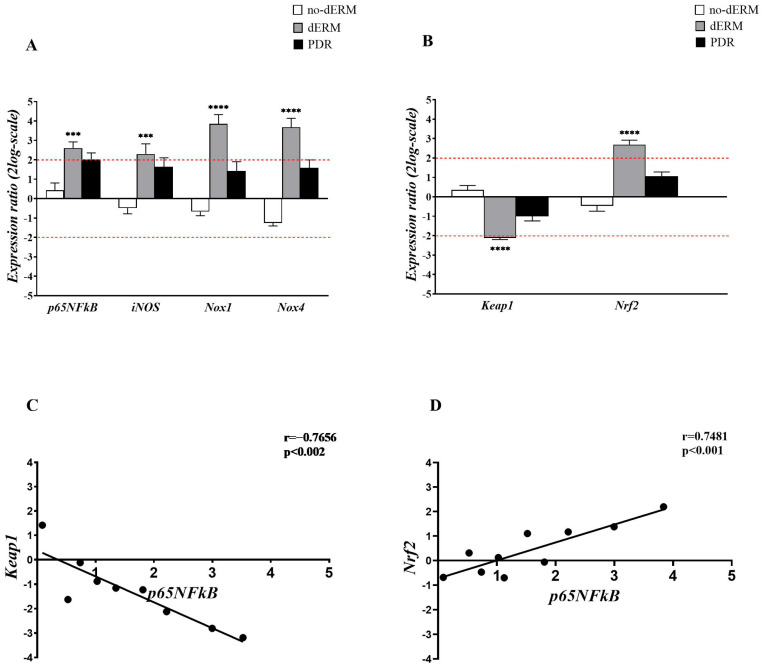
Inflammatory and oxidative stress regulators in aqueous cells. (**A**,**B**) Relative expression ratio (fold-changes) in no-dERM, dERM, and PDR patients with respect to controls. (**A**) Bar-graph showing a significant upregulation for *p65NFkB*, *iNOS*, *Nox1* and *Nox4* transcripts in dERM patients with respect to PDR, no-dERM, and controls. (**B**) A significant deregulation of *Keap1* and upregulation of *Nrf2* were observed in dERM with respect to PDR, no-dERM, and controls. Statistical assessment performed with the Rest-ANOVA Dunnett’s coupled analysis as reported: *** *p* < 0.001, **** *p* < 0.0001; data are shown as mean ± SD in a log2-scale and the limit 2 was used as referring threshold (red dashed line; (**A**,**B**)). (**C**,**D**) Plots showing the correlation between the inflammatory transcription factor *p65NFĸB* and the epigenetic *Keap1* (**C**) and *Nrf2* (**D**) genes. The correlation values from the Pearson’s rho test analysis are shown in the panels.

The expressions of *p65NFkB*, *iNOS*, *Nox1*, *Nox4*, as well as *Keap1* and *Nrf2* transcripts, were investigated in total RNA extracts from aqueous cells belonging to no-dERM, dERM, PDR, and control patients (Figure 2). The transcription activity was assessed by relative RT-PCR with respect to controls (CTR).

As shown in Figure 2A, a consistent upregulation of *p65NFkB*, *iNOS*, *Nox1*, and *Nox4* transcripts was observed in dERM compared with controls. The analysis of epigenetic targets showed opposite changes for *Keap1* and *Nrf2* transcripts in dERMs; a significant *Keap1mRNA* downregulation and a significant *Nrf2mRNA* upregulation were observed in dERM with respect to PDR, no-dERM, and controls (Figure 2B).

The Pearson’s rho test analysis pointed at *p65NFkB*−*Keap1* transcripts negative correlation (rho = −0.7656; *p* < 0.002; Figure 2C) and *p65NFk*−*Nrf2* positive correlation (rho = 0.7481; *p* < 0.001; Figure 2D). The *Keap1*−*Nrf2* parameters were negatively related (rho = −0.7027; *p* < 0.001).

### 3.2. Complement Receptors and Modulators Are Over-Expressed in Aqueous Cells from dERM

**Figure 3:**
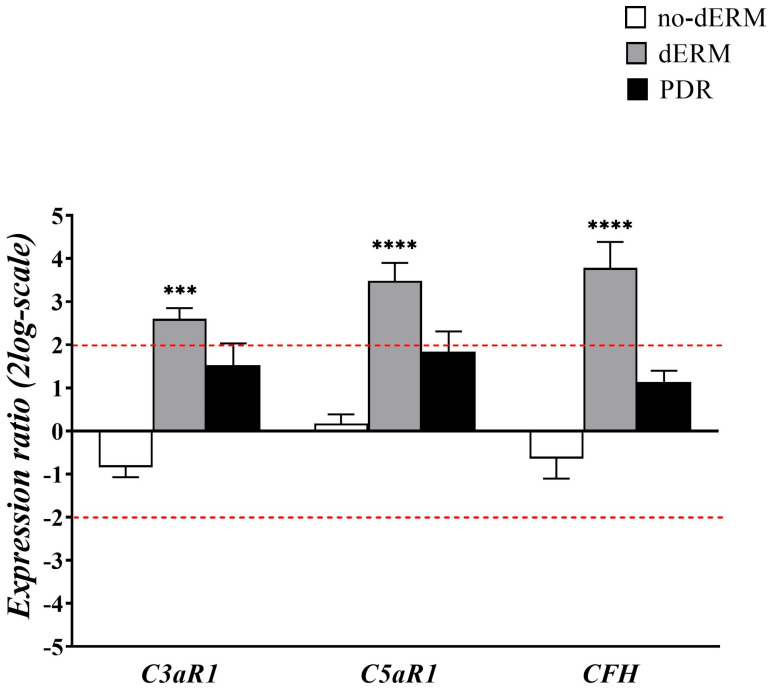
Complement receptors and modulator transcript expression in aqueous cells. The bar-graph shows the relative expression ratio (fold–changes) in no-dERM, dERM, and PDR with respect to controls. Note the significant upregulation for *C3aR1*, *C5aR1*, and *CFH* transcripts in dERM with respect to PDR, no-dERM, and controls. Data are shown as mean ± SD in log2-scale (threshold is indicated by red dashed line); rest-ANOVA Dunnett’s coupled analysis; *** *p* < 0.001, **** *p* < 0.0001.

As for oxidative stress regulators, transcripts’ expression of *C3a/C5a receptors1* and *complement factor H* were investigated in total RNAs from aqueous cells of no-dERM, dERM, PDR, and control patients (Figure 3).

Patients with dERM had significantly higher *C3aR1*, *C5aR1*, and *CFH* compared with PDR, no-dERM, and controls.

### 3.3. C3a, C5b9, and MDA Are Increased in dERM Vitreal Fluids

**Figure 4:**
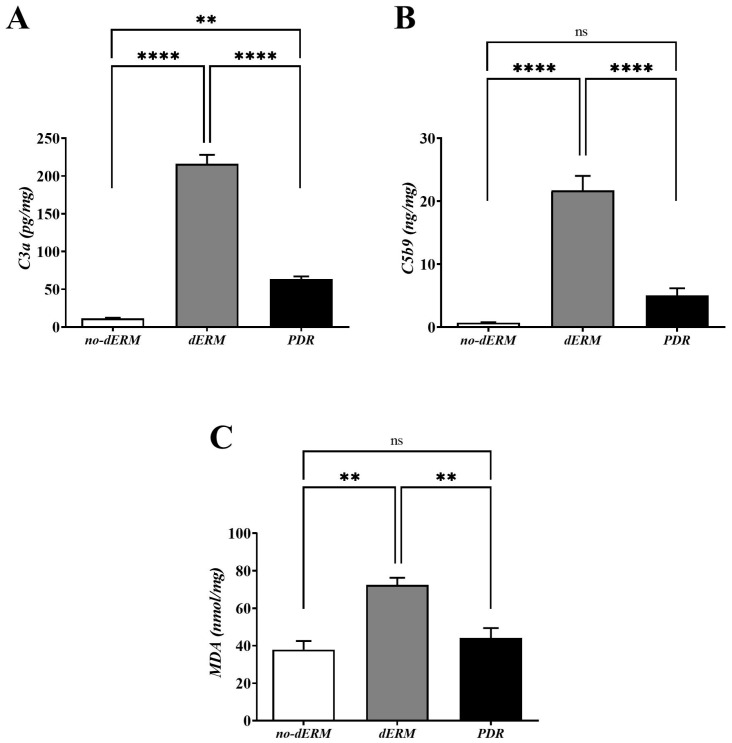
C3a, C5b9, and MDA levels in vitreous from no-dERM, dERM, and PDR patients. C3a (**A**), C5b9 (**B**) and MDA (**C**) levels were significantly increased in dERM vitreous with respect to PDR and no-dERM ones (C3a and C5b9, **** *p* < 0.0001; MDA, ** *p* < 0.01) as calculated by one-way ANOVA followed by a Tukey–Kramer post hoc test. Changes in C5b9 (**B**) and MDA (**C**) levels between PDR and no-dERMs were not significant (ns, *p* > 0.05). Data are shown as mean ± SD (C3a, pg/mg total protein; C5b9, ng/mg total protein; MDA, nmol/mg total protein).

The levels of C3a, C5b9, and MDA were quantified in vitreous by conventional ELISA (Figure 4). Higher C3a (Figure 4A) and C5b9 (Figure 4B) protein levels were observed in dERM vitreous, as compared with PDR and no-dERM ones (both *p* < 0.0001). Statistically significant changes were observed for C3a (*p* < 0.01; Figure 4A) but not for C5b9 (Figure 4B) in PDR vitreous as compared with no-dERM.

As shown in Figure 4C, the level of MDA was significantly high in the vitreous of dERM patients, as compared with PDR and no-dERM patients (*p* < 0.01). No change was observed for the levels of MDA in PDR compared with no-dERM.

## 4. Discussion

**Figure 5:**
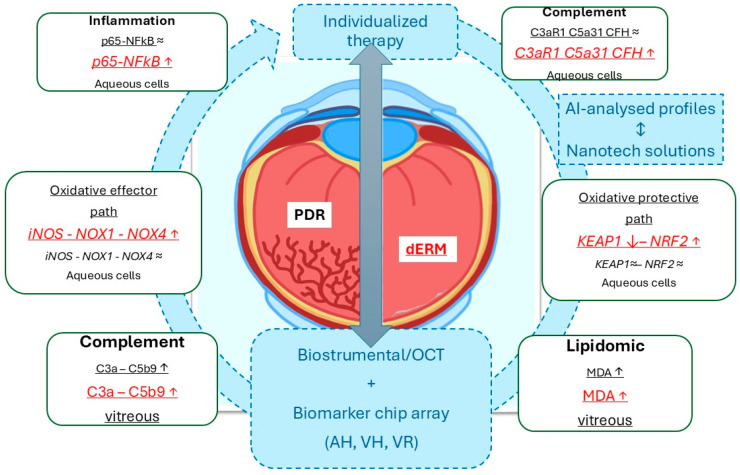
Schematic representation of the data obtained from this biomolecular analysis carried out on aqueous cells and clear vitreous from dERM (red) and PDR (black), both collected at the time of phaco-vitrectomy. As highlighted, the Keap1-Nrf2 signal pathway might suggest an opportunity to escape from disease progression, as observed in dERM rather than PDR. This path is one of the major regulators for cell protection against oxidative stress and complement dysregulation (ROS, RNS, complement fragments). Any possibility to stratify patients for an individualized therapy could be achievable by the crosstalk between biostrumental (including high-resolution imaging techniques) and biomolecular candidates. In this context, the artificial intelligence-driven biomarker selection for the preparation of nanotechnology-based ophthalmic drugs represents a mandatory step for support a tailored therapy. The gray ⬌ indicates the mandatory tools for individualized therapy. Biomarker expressions (inside rectangle dialog boxes) were reported in red for dERM and black for PDR, with arrows indicating upregulation/increase (↑) or downregulation/decrease (↓). The arrow-circle and the blue dotted squares indicate all the main aspects in the process for individualized therapy. Legend: AI, artificial intelligence; dERM, diabetic epiretinal membrane; PDR, proliferative diabetic retinopathy; OCT, optical coherence tomography; ROS, reactive oxigen species; RNS, reactive nitrogen species; AH, aqueous humor; VH, vitreous humor, VR, vitreal reflux.

By using vitreous and aqueous (cells) collected at the time of phaco-vitrectomy, this study highlighted that (i) *p65NFkB*, *iNOS*, and *Nox1/Nox4* were differentially expressed in PDR and dERM, highly expressed in dERM aqueous cells, and unexpressed in no-dERM; (ii) *Keap1* and *Nrf2* transcripts were inversely related in dERM aqueous cells and quite unchanged in both PDR and no-dERM; (iii) *C3aR1*, *C5aR1*, and *CFH* transcripts were overexpressed in dERM aqueous cells; and, finally, (iv) C3a, C5b9, and MDA levels were significantly increased in dERM vitreous, as summarized in Figure 5. The rationale behind this pattern of expression between oxidative, complement, and lipogenic factors is discussed below.

The diabetic form of VRD are caused principally by hyperglycemia, one of the major cellular effects leading to oxidative stress and complement cascade activation [6]. Systemic and local hyperglycemia induce the activation of NF-kB pathway, a key mediator of inflammatory responses [38,39,40]. Herein, we observed that *p65NFkB* transcripts were significantly overexpressed in dERM and slightly expressed in PDR, both with respect to no-dERM and controls. As is known, NF-kB drives the expression of proinflammatory cytokines (IL-1β and TNF-α), iNOS, ROS/RNS products, MAPK second messengers, as well as further activation of NF-kB, triggering physiological changes at cellular level (proliferation, differentiation, migration, survival, caspase-mediated apoptosis, and depolarization) [41,42]. Herein, we also observed an increased *iNOS* transcript expression in aqueous dERM cells with respect to no-dERM while no differences occurred with PDR. These data are in line with previous studies on the NF-kB ability to modulate the expression of the inducible isoform of NO synthase (iNOS) in diabetic human retina [43,44,45,46]. Since we found a significant upregulation for *Nox1* and *Nox4* transcripts in dERM with respect to PDR and an opposite trend in no-dERM, we can conclude that *Nox1* and *Nox4* can drive the impairments of retinal vascular permeability and promote the expression of inflammatory factors, as well as angiogenic factors in retinal diseases [47]. This is in line with the literature showing the involvement of iNOS in vascular changes associated with the early stages of DR, and the ability of Nox transcripts to activate Nox complex, enhancing oxidative stress and upregulating the expression of some enzymes responsible for mitochondrial impairment in DR (matrix metalloproteinases such as gelatinases) [48,49,50].

Some neuroprotective mechanisms can be attributed to the Keap1 and Nrf2, two key nuclear transcription factors involved in systemic and local antioxidant defense, including diabetes, DR, and likewise VRD [51]. The Keap1-Nrf2 signal pathway seems to be engaged in the regulation of the cellular response to oxidative stress by activating *Nrf2* [52]. Our analysis showed that the *Keap1*/*Nrf2* ratio is low in dERM, highlighting an overtranscription of *Nrf2* and sustaining the involvement of epigenetic pathways [52]. Under physiological non-oxidative circumstances, Keap1 anchors Nrf2 into cytoplasm to suppress its expression, while upon oxidative stress, ROS can change the conformation of Keap1 and decoupled Nrf2 can upregulate the specific gene expression (phase II detoxifying enzymes, antioxidant proteinase, and ubiquitinating enzymes) [51,53,54,55,56]. Our data regarding the significant upregulation of *Nrf2* expression in dERM with respect to PDR and no-dERM, and the significant deregulation of *Keap1* would indicate the potential activation of a protective endogenous oxidative stress system [51].

In retinal diseases, the relationship between oxidative stress and complement system activation is close and bidirectional, as oxidative stress can activate the complement pathway and vice versa [57]. The complement system is recognized as a key player in the pathogenesis of DR, and recent studies suggest an association between complement activation and DR progression, with greater involvement in PDR than NPDR, evaluating its potential as a biomarker of severity and therapeutic target [30,58,59,60]. Complement pathways work as the first line of innate immune defense throughout three separate pathways that in tandem promote inflammation, pathogen opsonization, cell membrane attack, and host immune response enhancement, and they are balanced by free-circulating regulators and receptors to prevent tissue damage [61,62]. As the eye is an immune-privileged tissue, circulating complement proteins are not able to move freely inside the neuronal retina when the BRB is intact, while under pathological states complement fragments can leak into the retinal parenchyma, causing and/or exacerbating the inflammatory profile [63]. Oxidative products from lipid peroxidation, proteins, and DNA fragmentation (oxidized lipids, carboxylated proteins, and DNA fragments) can be quickly recognized as “non-self” by the alternative complement pathway, which in turn allows quick tissue protection, even if the lost of the Complement Factor H (CFH) regulation, working in the prevention of excessive complement activation, can lead to further tissue damage and inflammation, such as under chronic inflammatory states [64,65]. In fact, the overproduction of C5a and C3a can stimulate the activation of microglia and macrophages, which in turn can release further ROS/RNS, causing sublethal damage to retinal cells and extensive intracellular ROS release, creating a vicious cycle, as observed in DR [66]. Corroborating data sustain the activation of intraocular C3 and C5 fragments and their related C3a/C3 and C5a/C5 ratios in PDR, as well as the concomitant upregulation of C3bα’ fragments and CFH in activated microglia in the PDR vitreous chamber [32]. A definitive role for the alternative complement pathway in DR pathogenesis was provided by the analysis of CFB and CFH [32].

Herein we focused the attention on a few complement receptors and modulators (*C3aR1*, *C5aR1*, and *CFH*) that were found statistically upregulated in dERM aqueous cells with respect to PDR and no-dERM. This increased expression might reflect a chronic inflammatory response characterized by complement regulatory mechanisms that remain more active and functional in dERM with respect to PDR and no-dERM. The different regulations of the complement system might be explained by the loss of complement homeostatic control in PDR, probably due to overt hypoxia, long-lasting inflammation, and impairment of complement regulators (CFH) [32]. The herein reported upregulation of *CFH* transcripts could suggest a feedback mechanism for arresting excessive complement activation, according to the fact that CFH is a negative regulator of the alternative complement path, although it can also be associated with a dysregulation of the system [32,67].

Ocular humors and their related compartments have a different protein turnover, as vitreous (posterior chamber) is more static and accumulates inflammatory as well as pro-oxidant proteins, while aqueous (anterior chamber) reacts with an active systemic and local turn-over [68]. As well as different cellular patterns characterize both fluids under physiological and pathological conditions [68]. Our studies indicate an increased expression of C3a, C5b9, and MDA accumulation in the vitreous of dERM with respect to PDR and no-dERM, confirming the involvement of the complement and oxidative (lipidic) pathways also in the posterior chamber [67]. MDA represents the most widely used biomarker for evaluating oxidative stress at cellular level and monitoring the efficacy of an antioxidant retinal therapy, if the access to vitreous is available [25,26,27,28,29].

Finally, the following two major limits can be identified in this observational protocol: i. a small size of the study population due to the stringency used to select patient according to exclusion criteria and ii. the absence of data on the quantification of complement fragments (C3a and C5b9) and MDA in clear aqueous due to the limited volume of humor at the time of phaco-vitrectomy to carry out conventional ELISA assays.

## 5. Conclusions

The retina displays its own unique immune regulatory mechanisms and immune defense mechanisms adopted by the local glia and microglia populations [69]. The retinal immune defense mechanism is alerted by any kind of noxious signal, including insults leading to oxidative stress, complement activation, and lipidomic metabolism, and starts a peculiar innate and adaptive response to restore the homeostatic balance (parainflammation) [69,70]. Low-level activation of the complement system is required to preserve normal vitreous chamber homeostasis and maintain retinal integrity with aging [32]. Under physiological conditions, these paths are deregulated or tightly regulated, while in several ocular pathologies, uncontrolled activation can drive the disease’s progression [69]. At the early stages of disease, when the blood–retinal barrier (BRB) is still intact, the retinal microglia and the entire complement pathway are activated at low levels of inflammation (parainflammation), retaining both homeostasis and functionality (protective mechanism) [69,70]. On the other side, a detrimental long-lasting (physical, mechanical, biological) insult can lead to an irreversible loss of functionality, as observed in some ocular neurodegenerative diseases (age-related macular degeneration, DR, AD, and PD [69,70,71]. In PDR and dERM, the complement activation occurs in different routes and specific areas, and this increased complement activation can trigger the photoreceptor cell death and consequent retinal detachment, highlighting the impact of the complement system in various retinal pathologies [32]. Our findings on the transcript expression of complement receptors—between *C3aR1*, *C5aR1*, and *CFH*—in aqueous cells have raised interesting questions on the role of these mediators in PDR and dERM progression and severity. On the other side, a contribution of the Keap1/Nrf2 path in the mitigation of oxidative stress-induced complement activation with subsequent modulatory effects of cell damage can also be hypothesized. In fact, both *C3aR1*, *C5aR1*, and *CFH* as well as *Nrf2*, seem to be activated in dERM aqueous cells, in parallel with C3a, C5b9, and MDA accumulation in the vitreous, providing potential implications in the modulation of parainflammation and/or inflammageing in dERM with respect to PDR [72]. These findings would imply that dERM aqueous cells are activated upon hyperglycemia and in the presence of epiretinal membranes, and this activation can cause significant oxidative stress and lipidomic effects that might trigger cellular dysfunction and deposition of complement-stimulating proteins on receptors paralleling the vitreal accumulation.

Taken together, these findings suggest the involvement of oxidative stress, lipidomic metabolism, and complement activation in dERM and PDR, although most probably with different dynamics. Our findings, supported by the connection between the intraocular chambers and the possibility that aqueous can mirror the vitreous protein signature, open to the consideration of the potential therapeutic intervention using different routes of ocular drug delivery (monoclonal antibodies, Au@pCH NPs, implants, liposomes), based on the protein profile analysis coupled to artificial intelligence. This aspect might represent a step forward in the treatment of VRD, DR, and macular degenerative diseases, as observed in other disciplines in the case of individualized therapies (see Figure 5) [68,72,73,74,75]. In this context, the loading of specific antioxidant drugs and/or complement inhibitors (anti-C5 antibodies) cannot be excluded for interrupting the vicious oxidative stress-complement damaging circuit, in addition to the anti-angiogenic mediators (anti-VEGF and anti-Ang2), as multiple therapeutic approaches in the “era” of precision medicine.

## Figures and Tables

**Table 1:** Study population.

	CTR ^a^	no-dERM ^b^	dERM ^c^	PDR ^d^
**Eyes/patients**	6	9	6	6
**Gender (M/F)**	3M/3F	4M/5F	3M/3F	5M/1F
**Age ^e^**	77.7 ± 9.0	70.4 ± 6.4	73.2 ± 4.9	63.7 ± 7.4

^a^ Cataract; ^b^ no-diabetic epiretinal membrane; ^c^ diabetic epiretinal membrane; ^d^ proliferative diabetic retinopathy having ERM; ^e^ mean ± SD.

**Table 2:** Primer description.

Genes	Primers Pairs		Accession Number
**Referring Genes**			
*H3*	F: GCTTCGAGAGATTCGTCGTT	R: GAAACCTCAGGTCGGTTTTG	NM_005324
**Target Genes**			
*p65NFkB*	F: CAGAAGCAGGCTGGAGGTAA	R: GTTAGGCACAGGGACAATGC	L19067.1
*iNOS*	F: CCCCTTCAATGGCTGGTACA	R: GTTTCCAGGCCCATTCTCCT	U31511.1
*Nox1*	F: CCAGGATTGAAGTGGATGGT	R: AGGTTGTGGTCTGCACACTG	BC075014.2
*Nox4*	F: CTCAGCGGAATCAATCAGCTGTG	R: AGAGGAACACGACAATCAGCCTTAG	BC040105.1
*C3aR1*	F: TATGCAAGCTCATCCCCTCCAT	R: TGCACATCACAAAAGCCACCA	BC020742.1
*C5aR1*	F: TGGCCTTGGTCATCTTTGCA	R: GATGCTGTACAATGGACGTGAAC	BC008982.1
*CFH*	F: TTGCACACAAGATGGATGGTCG	R: CATGTAACTGTGGTCTGCGCTT	Y00716.1
*Keap1*	F: TTCAGCTACACCCTGGAGGA	R: CTTGAAGACAGGGCTGGATG	BC002417.2
*Nrf2*	F: ACACGGTCCACAGCTCATC	R: TGCCTCCAAAGTATGTCAATCA	BC011558.1

## Data Availability

All data generated or analyzed during this study are included in this published article.
